# Elliptic Solutions of Dynamical Lucas Sequences

**DOI:** 10.3390/e23020183

**Published:** 2021-01-31

**Authors:** Michael J. Schlosser, Meesue Yoo

**Affiliations:** 1 Fakultät für Mathematik, Universität Wien, Oskar-Morgenstern-Platz 1, A-1090 Vienna, Austria; 2 Department of Mathematics, Chungbuk National University, Cheongju 28644, Korea; meesueyoo@chungbuk.ac.kr

**Keywords:** Lucas sequences, theta functions, elliptic numbers, non-commutative Fibonacci polynomials

## Abstract

We study two types of dynamical extensions of Lucas sequences and give elliptic solutions for them. The first type concerns a level-dependent (or discrete time-dependent) version involving commuting variables. We show that a nice solution for this system is given by elliptic numbers. The second type involves a non-commutative version of Lucas sequences which defines the non-commutative (or abstract) Fibonacci polynomials introduced by Johann Cigler. If the non-commuting variables are specialized to be elliptic-commuting variables the abstract Fibonacci polynomials become non-commutative elliptic Fibonacci polynomials. Some properties we derive for these include their explicit expansion in terms of normalized monomials and a non-commutative elliptic Euler–Cassini identity.

## 1. Introduction

In a series of papers, Lucas [1,2,3] studied the generalized Fibonacci polynomials 〈n〉 which depend on two commuting variables P,Q and are defined by 〈0〉=0, 〈1〉=1, and

(1) 〈n〉=P〈n−1〉+Q〈n−2〉,

for n≥2. (The two initial conditions 〈0〉=0, 〈1〉=1 can be altered of course but we shall stick to them here as specified.) For example, we have

$$\langle 2\rangle =P,\;\langle 3\rangle =P^{2}+Q,\;\langle 4\rangle =P^{3}+2PQ,\;\langle 5\rangle =P^{4}+3P^{2}Q+Q^{2}.$$

For P=Q=1, this sequence reduces to the Fibonacci numbers $\langle n\rangle =F_{n}$. For P=2, Q=−1 it reduces to the nonnegative integers 〈n〉=n. For $P=q+q^{-1}$, Q=−1, it reduces to the quantum integers $\langle n\rangle ={\langle n\rangle }_{q}:=\frac{q^{n}-q^{-n}}{q-q^{-1}}$, while for P=1+q, Q=−q, it reduces to the (standard) *q*-integers $\langle n\rangle ={\left[n\right]}_{q}:=\frac{1-q^{n}}{1-q}$. More generally, for P=c(1+q) and $Q=-c^{2}q$, it reduces to $c^{n-1}{\left[n\right]}_{q}$, unifying the last two cases.

A function is defined to be *elliptic* if it is meromorphic and doubly periodic. It is well known (cf., e.g., [4]) that elliptic functions can be expressed in terms of quotients of products of theta functions. Define for z≠0 the *(modified Jacobi) theta function* with *nome* *p* by

$$\theta \left(z;p\right)=\prod_{j\geq 0}\left(\left(1-p^{j}z\right)\left(1-p^{j+1}/z\right)\right),\;\;\left|p\right|<1.$$

For brevity, we write

$$\theta \left(z_{1},\cdots ,z_{m};p\right)=\theta \left(z_{1};p\right)\cdots \theta \left(z_{m};p\right)$$

for products of these functions. The modified Jacobi theta functions satisfy the *inversion formula*

(2a) θ(z;p)=−zθ(1/z;p),

the *quasi-periodicity relation*

(2b) $$\theta \left(pz;p\right)=-\frac{1}{z}\theta \left(z;p\right),$$

and the *addition formula*

(2c) $$\theta \left(uv,u/v,wz,w/z;p\right)-\theta \left(uz,u/z,wv,w/v;p\right)=\frac{w}{v}\;\theta \left(vz,v/z,uw,u/w;p\right)$$

(cf. p. 451, Example 5 in [5]).

In this paper, we study two types of dynamical extensions of Lucas sequences and give *elliptic* solutions for them. The first type concerns a *level-dependent* (or *discrete time-dependent*) version of (1) involving commuting variables. We show that a nice solution for this system is given in terms of elliptic numbers. The second type is a *non-commutative* version which defines the *non-commutative* (or *abstract*) *Fibonacci polynomials* introduced by Johann Cigler [6]. We study some (known and new) properties for these. In particular, we extend the sequence of these polynomials to negative indices and recover a formula by Cigler (Section 3 in [7]) for the negatively indexed non-commutative Fibonacci polynomials in terms of the non-negatively indexed ones. This allows us to establish a non-commutative Euler–Cassini identity. In the non-commutative setting we also take a closer look at the case when the non-commuting variables are specialized to satisfy *weight-dependent commutation relations*. In this case the non-commutative Fibonacci polynomials become, what we shall call, *non-commutative weight-dependent Fibonacci polynomials*. We show that after normal ordering of the weight-dependent-commuting variables weight-dependent binomial coefficients appear in the expansion of the normalized monomials. A further specialization of interest concerns the introduction of elliptic weights. For *elliptic-commuting variables* the non-commutative Fibonacci polynomials become, what we shall call, *non-commutative elliptic Fibonacci polynomials*. In this case after normal ordering of the elliptic-commuting variables fully factorized elliptic binomial coefficients appear in the expansion of the normalized monomials. This extends the basic case (or *q*-case) for *q*-commuting variables. We also establish an explicit Euler–Cassini identity for the non-commutative elliptic Fibonacci polynomials.

We would like to point out that the results in the current paper do not appear to directly contain the *elliptic Fibonacci numbers* which were introduced in [8] nor those (of a simpler type) which were introduced in [9]. While we believe that there is a connection of our non-commutative elliptic Fibonacci polynomials considered in Section 4 of this paper with our earlier elliptic Fibonacci numbers in [8], the connection is not yet entirely clear and requires further investigations.

Our paper is organized as follows. In Section 2 we study the level-dependent Lucas system with commutative variables and give an elliptic solution for it. In Section 3 we describe the algebras of weight-dependent-commuting and elliptic-commuting variables we are working with in the final section, and also define corresponding weighted and elliptic binomial coefficients. Finally, Section 4 is devoted to the non-commutative Lucas equation and the noncommutative weight-dependent and elliptic Fibonacci polynomials.

## 2. Elliptic Solution of a Level-Dependent Lucas System

In this section, we consider the following level-dependent extension of Lucas’ generalized Fibonacci polynomials 〈n〉 defined by the recurrence relation (1). We consider sequences of variables ${\left(P_{\ell }\right)}_{\ell \geq 0}$ and ${\left(Q_{\ell }\right)}_{\ell \geq 0}$ (where the index *ℓ* could be thought of being the *level* or *discrete time*). Now define the doubly-indexed sequence ${\left({\langle n\rangle }_{\ell }\right)}_{n,\ell \geq 0}$ by ${\langle 0\rangle }_{\ell }=0$, ${\langle 1\rangle }_{\ell }=1$, for all ℓ≥0 and, instead of (1), assume the following dynamical recurrence relation:

(3) $${\langle n\rangle }_{\ell }=P_{\ell }{\langle n-1\rangle }_{\ell +1}+Q_{\ell }{\langle n-2\rangle }_{\ell +2},$$

for n≥2 and all ℓ≥0. Here we have

$$\begin{array}{cc} {\langle 2\rangle }_{\ell } & =P_{\ell }\\ {\langle 3\rangle }_{\ell } & =P_{\ell }P_{\ell +1}+Q_{\ell },\\ {\langle 4\rangle }_{\ell } & =P_{\ell }P_{\ell +1}P_{\ell +2}+P_{\ell }Q_{\ell +1}+P_{\ell +2}Q_{\ell },\\ {\langle 5\rangle }_{\ell } & =P_{\ell }P_{\ell +1}P_{\ell +2}P_{\ell +3}+P_{\ell }P_{\ell +1}Q_{\ell +2}+P_{\ell }P_{\ell +3}Q_{\ell +1}+P_{\ell +2}P_{\ell +3}Q_{\ell }+Q_{\ell }Q_{\ell +2}, \end{array}$$

for all l≥0.

We now show that the system in (3) admits a nice solution involving elliptic functions.

Let *a* and *b* be two independent variables, and q∈C be the *base*. It readily follows by the addition formula (2c) that for

(4) $$P_{\ell }=\frac{\theta (q^{2},aq^{\ell +2},bq^{2\ell +2},aq^{-\ell }/b;p)}{\theta (q,aq^{\ell +1},bq^{2\ell +3},aq^{1-\ell }/b;p)},\;\;Q_{\ell }=-\frac{\theta (aq^{\ell +3},bq^{2\ell +1},aq^{-1-\ell }/b;p)}{\theta (aq^{\ell +1},bq^{2\ell +3},aq^{1-\ell }/b;p)}q$$

the sequence defined by the system in (3) reduces to the *elliptic integers*

(5) $${\langle n\rangle }_{\ell }={\langle n\rangle }_{aq^{\ell },bq^{2\ell };q,p}:=\frac{\theta (q^{n},aq^{\ell +n},bq^{2\ell +n},aq^{2-\ell -n}/b;p)}{\theta (q,aq^{\ell +1},bq^{2\ell +2n-1},aq^{1-\ell }/b;p)}.$$

Indeed, if we insert $P_{\ell }$ and $Q_{\ell }$ from (4) in (3), we obtain

$$\begin{array}{cc} \; & P_{\ell }{\langle n-1\rangle }_{\ell +1}+Q_{\ell }{\langle n-2\rangle }_{\ell +2}\\ \; & =\frac{\theta (q^{2},aq^{\ell +2},bq^{2\ell +2},aq^{-\ell }/b;p)}{\theta (q,aq^{\ell +1},bq^{2\ell +3},aq^{1-\ell }/b;p)}\;\frac{\theta (q^{n-1},aq^{\ell +n},bq^{2\ell +n+1},aq^{2-\ell -n}/b;p)}{\theta (q,aq^{\ell +2},bq^{2\ell +2n-1},aq^{-\ell }/b;p)}\\ \; & \;\;-\frac{\theta (aq^{\ell +3},bq^{2\ell +1},aq^{-1-\ell }/b;p)}{\theta (aq^{\ell +1},bq^{2\ell +3},aq^{1-\ell }/b;p)}q\;\frac{\theta (q^{n-2},aq^{\ell +n},bq^{2\ell +n+2},aq^{2-\ell -n}/b;p)}{\theta (q,aq^{\ell +3},bq^{2\ell +2n-1},aq^{-1-\ell }/b;p)}\\ \; & =\frac{\theta (q^{2},bq^{2\ell +2},q^{n-1},aq^{\ell +n},bq^{2\ell +n+1},aq^{2-\ell -n}/b;p)}{\theta (q,aq^{\ell +1},bq^{2\ell +3},aq^{1-\ell }/b,q,bq^{2\ell +2n-1};p)}\\ \; & \;\;-q\frac{\theta (bq^{2\ell +1},q^{n-2},aq^{\ell +n},bq^{2\ell +n+2},aq^{2-\ell -n}/b;p)}{\theta (aq^{\ell +1},bq^{2\ell +3},aq^{1-\ell }/b,q,bq^{2\ell +2n-1};p)}\\ \; & =\frac{\theta (aq^{\ell +n},aq^{2-\ell -n}/b;p)}{\theta (q,aq^{\ell +1},bq^{2\ell +3},aq^{1-\ell }/b,q,bq^{2\ell +2n-1};p)}\\ \; & \;\;\times \left[\theta \left(q^{2},bq^{2\ell +2},q^{n-1},bq^{2\ell +n+1};p\right)-q\;\theta \left(q,bq^{2\ell +1},q^{n-2},bq^{2\ell +n+2};p\right)\right]\\ \; & =\frac{\theta (aq^{\ell +n},aq^{2-\ell -n}/b;p)}{\theta (q,aq^{\ell +1},bq^{2\ell +3},aq^{1-\ell }/b,q,bq^{2\ell +2n-1};p)}\theta \left(q^{n},bq^{2\ell +n},q,bq^{2\ell +3};p\right)\\ \; & =\frac{\theta (q^{n},aq^{\ell +n},bq^{2\ell +n},aq^{2-\ell -n}/b;p)}{\theta (q,aq^{\ell +1},bq^{2\ell +2n-1},aq^{1-\ell }/b;p)}={\langle n\rangle }_{\ell }, \end{array}$$

where the difference of the two products of theta functions in the pair of brackets in the fourth equality was simplified with respect to the

$$\left(u,v,w,z\right)\mapsto \left(b^{\frac{1}{2}}q^{\ell +n},b^{\frac{1}{2}}q^{\ell +1},b^{\frac{1}{2}}q^{\ell +2},b^{\frac{1}{2}}q^{\ell }\right)$$

case of (2c). This proves the claim about the elliptic solution.

The elliptic integers in (5) can actually be identified as specialized elliptic binomial coefficients

$${\langle n\rangle }_{\ell }={\begin{array}{c} \left[\begin{array}{c} n\\ n-1 \end{array}\right] \end{array}}_{aq^{\ell },bq^{2\ell };q,p},$$

the general case of the elliptic binomial coefficients being defined in (16).

Finally, we point out a simple way to obtain a new dynamical Lucas sequence from a given one by a suitable “scaling” of the variables with respect to an additional sequence ${\left(c_{\ell }\right)}_{\ell \geq 0}$. In particular, given three sequences

$${\left({\langle n\rangle }_{\ell }\right)}_{n,\ell \geq 0},\;{\left(P_{\ell }\right)}_{\ell \geq 0},\;{\left(Q_{\ell }\right)}_{\ell \geq 0},$$

satisfying (3) with the initial conditions ${\langle 0\rangle }_{\ell }=0$ and ${\langle 1\rangle }_{\ell }=1$, for all ℓ≥0, the three sequences

$${\left({\tilde{\langle n\rangle }}_{\ell }\right)}_{n,\ell \geq 0},\;{\left({\tilde{P}}_{\ell }\right)}_{\ell \geq 0},\;{\left({\tilde{Q}}_{\ell }\right)}_{\ell \geq 0},$$

with the initial conditions ${\tilde{\langle 0\rangle }}_{\ell }=0$ and ${\tilde{\langle 1\rangle }}_{\ell }=1$, for all ℓ≥0, also satisfy (3), where

$${\tilde{\langle n\rangle }}_{\ell }=c_{\ell }c_{\ell +1}\cdots c_{\ell +n-2}{\langle n\rangle }_{\ell },\;{\tilde{P}}_{\ell }=c_{\ell }P_{\ell },\;{\tilde{Q}}_{\ell }=c_{\ell }c_{\ell +1}Q_{\ell },$$

for all n≥2 and ℓ≥0. It is straightforward to confirm this assertion by multiplying both sides of (3) with the product $c_{\ell }c_{\ell +1}\cdots c_{\ell +n-2}$.

## 3. Weight-Dependent Commutation Relations and Elliptic Weights

### 3.1. Noncommutative Weight-Dependent Binomial Theorem

The material in this subsection, up to Lemma 1, is taken from the first author’s paper [10], while the material afterwards is new.

Let N and $N_{0}$ denote the sets of positive and nonnegative integers, respectively.

**Definition** **1.** *For a doubly-indexed sequence of indeterminates ${\left(w\left(s,t\right)\right)}_{s,t\in N}$, let $C_{w}\left[x,y\right]$ be the associative unital algebra over C generated by x and y, satisfying the following three relations:*

(6a) $$\begin{array}{cc} yx & =w(1,1)xy, \end{array}$$

(6b) $$\begin{array}{cc} xw(s,t) & =w(s+1,t)x, \end{array}$$

(6c) $$\begin{array}{cc} yw(s,t) & =w(s,t+1)y, \end{array}$$

*for all s,t∈N.*

For s∈N and $t\in N_{0}$, we define

(7) $$W\left(s,t\right):=\prod_{j=1}^{t}w\left(s,j\right),$$

the empty product being defined to be 1. Note that for s,t∈N, we have w(s,t)=W(s,t)/W(s,t−1). We refer to the w(s,t) as *small weights*, whereas to the W(s,t) as *big weights* (or *column weights*).

Let the *weight-dependent binomial coefficients* be defined by

(8a) www00=1,wwwnk=0forn∈N0,andk∈−Nork>n,

and

(8b) 1−1wwwn+1k=wwwnk+wwwnk−1W(k,n+1−k)forn,k∈N0.

These weight-dependent binomial coefficients have a combinatorial interpretation in terms of *weighted lattice paths*, see [11]. Here, a lattice path is a sequence of north (or vertical) and east (or horizontal) steps in the first quadrant of the xy-plane, starting at the origin (0,0) and ending at say (n,m). We give weights to such paths by assigning the big weight W(s,t) to each east step (s−1,t)→(s,t) and 1 to each north step. Then define the weight of a path *P*, w(P), to be the product of the weight of all its steps.

Given two points $A,B\in N_{0}^{2}$, let P(A→B) be the set of all lattice paths from *A* to *B*, and define

$$w\left(P\left(A\to B\right)\right):=\sum_{P\in P(A\to B)}w\left(P\right).$$

Then we have

(9) w(P((0,0)→(k,n−k)))=wwwnk

as both sides of the equation satisfy the same recursion and initial condition as in (Section 3.1).

Interpreting the *x*-variable as an east step and the *y*-variable as a north step, we get the following weight dependent binomial Theorem 1.

**Theorem** **1** ([10])**.** *Let $n\in N_{0}$. Then, as an identity in $C_{w}\left[x,y\right]$,*

(10) (x+y)n=∑k=0nwwwnkxkyn−k.

The following rule for interchanging powers of *x* and *y* is easy to prove by induction (and it is also easy to interpret combinatorially by considering weighted lattice paths); we therefore omit the proof.

**Lemma** **1** ([10])**.** *We have*

$$y^{k}x^{\ell }=\left(\prod_{i=1}^{\ell }\prod_{j=1}^{k}w\left(i,j\right)\right)x^{\ell }y^{k}=\left(\prod_{i=1}^{\ell }W\left(i,k\right)\right)x^{\ell }y^{k}.$$

We now extend the algebra $C_{w}\left[x,y\right]$ from Definition 1 to the algebra $C_{w}\left[x,x^{-1},y\right]$:

**Definition** **2.** *For a doubly-indexed sequence of invertible indeterminates ${\left(w\left(s,t\right)\right)}_{s\in Z,t\in N}$, let $C_{w}\left[x,x^{-1},y\right]$ be the associative unital algebra over C generated by x, $x^{-1}$ and y, satisfying the following relations:*

(11a) $$\begin{array}{cc} x^{-1}x & =xx^{-1}=1 \end{array}$$

(11b) $$\begin{array}{cc} yx & =w(1,1)xy, \end{array}$$

(11c) $$\begin{array}{cc} x^{-1}y & =w\left(0,1\right)yx^{-1}, \end{array}$$

(11d) $$\begin{array}{cc} xw(s,t) & =w(s+1,t)x, \end{array}$$

(11e) $$\begin{array}{cc} x^{-1}w\left(s,t\right) & =w\left(s-1,t\right)x^{-1}, \end{array}$$

(11f) $$\begin{array}{cc} yw(s,t) & =w(s,t+1)y, \end{array}$$

*for all s∈Z and t∈N.*

It is easy to see that the above relations are compatible with each other and naturally extend (6).

The following Lemma 2 which is easy to verify will be used in Section 4.

**Lemma** **2.** *Let ${\left(w\left(s,t\right)\right)}_{s\in Z,t\in N}$ be a doubly-indexed sequence of invertible indeterminates, and x and y variables with x being invertible, together forming the associative algebra $A=C_{w}\left[x,x^{-1},y\right]$.Then there is an involutive algebra isomorphism*

$$\phi :A\to \tilde{A}$$

*where*

$$\tilde{A}=C_{\tilde{w}}\left[x^{-1},x,x^{-1}y\right]$$

*with*

(12) $$\tilde{w}\left(s,t\right)=w{\left(1-s-t,t\right)}^{-1}.$$

It is indeed straightforward to check that the simultaneous replacement of w(s,t) (s∈Z, t∈N), *x* and *y* in (2) by $w{\left(1-s-t,t\right)}^{-1}$, $x^{-1}$ and $x^{-1}y$, respectively, again satisfies the conditions in (11).

As a consequence, given an identity in w(s,t) (s∈Z, t∈N), *x* and *y*, a new valid identity can be obtained by applying the isomorphism ϕ to each of the occurring variables, where in both identities the variables satisfy the same commutation relations (11).

### 3.2. Elliptic Weights

For nome p∈C with |p|<1, *base*q∈C, two independent variables *a* and *b*, and $\left(s,t\right)\in Z^{2}$, we define the *small elliptic weights* to be

(13a) $$w_{a,b;q,p}\left(s,t\right)=\frac{\theta (aq^{s+2t},bq^{2s+t-2},aq^{t-s-1}/b;p)}{\theta (aq^{s+2t-2},bq^{2s+t},aq^{t-s+1}/b;p)}q,$$

and the *big elliptic weights* to be

(13b) $$W_{a,b;q,p}\left(s,t\right)=\frac{\theta (aq^{s+2t},bq^{2s},bq^{2s-1},aq^{1-s}/b,aq^{-s}/b;p)}{\theta (aq^{s},bq^{2s+t},bq^{2s+t-1},aq^{t-s+1}/b,aq^{t-s}/b;p)}q^{t}.$$

Notice that for t≥0 we have

$$W_{a,b;q,p}\left(s,t\right)=\prod_{k=1}^{t}w_{a,b;q,p}\left(s,k\right).$$

Observe that

(14a) $$w_{a,b;q,p}\left(s+i,t+j\right)=w_{aq^{i+2j},bq^{2i+j};q,p}\left(s,t\right),$$

and

(14b) $$W_{a,b;q,p}\left(s,t+j\right)=W_{a,b;q,p}\left(s,j\right)\;W_{aq^{2j},bq^{j};q,p}\left(s,t\right),$$

for all *s*, *t*, *i* and *j*, which are elementary identities we will make use of.

Further, using (2a), we see directly from (13a) that

(15) $$w_{a,b;q,p}{\left(1-s-t,t\right)}^{-1}=w_{a/b,1/b;q,p}\left(s,t\right),$$

which can be conveniently applied when using Lemma 2.

The terminology “elliptic” for the above small and big weights is indeed justified, as the small weight $w_{a,b;q,p}\left(s,k\right)$ (and also the big weight) is elliptic in each of its parameters (i.e., these weights are even “totally elliptic”). Writing $q=e^{2\pi i\sigma }$, $p=e^{2\pi i\tau }$, $a=q^{\alpha }$ and $b=q^{\beta }$ with complex σ, τ, α, β, *s* and *k*, then the small weight $w_{a,b;q,p}\left(s,k\right)$ is clearly periodic in α with period $\sigma^{-1}$. Also, using (2b), we can see that $w_{a,b;q,p}\left(s,k\right)$ is also periodic in α with period $\tau \sigma^{-1}$. The same applies to $w_{a,b;q,p}\left(s,k\right)$ as a function in β (or *s* or *k*) with the same two periods $\sigma^{-1}$ and $\tau \sigma^{-1}$.

Next, we define (cf. Chapter 11 in [12]) the *theta shifted factorial* (or *q,p-shifted factorial*), by

$${\left(a;q,p\right)}_{n}=\left\{\begin{array}{cc} \prod_{j=0}^{n-1}\theta \left(aq^{j};p\right), & n=1,2,\ldots \;,\\ 1, & n=0,\\ 1/\prod_{j=0}^{-n-1}\theta \left(aq^{n+j};p\right), & n=-1,-2,\ldots , \end{array}\right.$$

and write

$${\left(a_{1},\ldots ,a_{m};q,p\right)}_{n}={\left(a_{1};q,p\right)}_{n}\ldots {\left(a_{m};q,p\right)}_{n},$$

for their products. For p=0 we have θ(x;0)=1−x and, hence, ${\left(a;q,0\right)}_{n}={\left(a;q\right)}_{n}=\left(1-a\right)\left(1-aq\right)\cdots \left(1-aq^{n-1}\right)$ is a *q-shifted factorial* in base *q*.

Now, the *elliptic binomial coefficients* [10]

(16) $${\left[\begin{array}{c} n\\ k \end{array}\right]}_{a,b;q,p}:=\frac{{\left(q^{1+k},aq^{1+k},bq^{1+k},aq^{1-k}/b;q,p\right)}_{n-k}}{{\left(q,aq,bq^{1+2k},aq/b;q,p\right)}_{n-k}},$$

together with the big elliptic weights defined in (13b), can be seen to satisfy the recursion (8), as a consequence of the addition formula (2c).

Note that the elliptic binomial coefficients in (16) generalize the familiar *q*-binomial coefficients, which can be obtained by letting p→0, a→0, then b→0, in this order. These are defined by

$${\left[\begin{array}{c} n\\ k \end{array}\right]}_{q}:=\frac{{\left(q^{1+k};q\right)}_{n-k}}{{\left(q;q\right)}_{n-k}},$$

where

$${\left(a;q\right)}_{n}=\left\{\begin{array}{cc} \prod_{j=0}^{n-1}\left(1-aq^{j}\right), & n=1,2,\ldots \;,\\ 1, & n=0,\\ 1/\prod_{j=0}^{-n-1}\left(1-aq^{n+j}\right), & n=-1,-2,\ldots \end{array}\right.$$

are the *q*-shifted factorials.

As the *q*-binomial coefficients satisfy two recurrence relations

$${\left[\begin{array}{c} n+1\\ k \end{array}\right]}_{q}={\left[\begin{array}{c} n\\ k \end{array}\right]}_{q}+{\left[\begin{array}{c} n\\ k-1 \end{array}\right]}_{q}q^{n+1-k},\;{\left[\begin{array}{c} n+1\\ k \end{array}\right]}_{q}={\left[\begin{array}{c} n\\ k \end{array}\right]}_{q}q^{k}+{\left[\begin{array}{c} n\\ k-1 \end{array}\right]}_{q},$$

and the recurrence relation (8) corresponds to the first identity, the elliptic binomial coefficients satisfy a second recurrence relation as well. While the relation (8) is established by considering the generating function of all weighted paths from the origin to the point (k,n+1−k) and separating them into two subsets depending on whether the last step is vertical or horizontal, the following result can be similarly verified by separating the same set of paths into two subsets depending on whether the first step is vertical or horizontal.

**Proposition** **1.** *We have*

$${\left[\begin{array}{c} n+1\\ k \end{array}\right]}_{a,b;q,p}={\left[\begin{array}{c} n\\ k \end{array}\right]}_{aq^{2},bq;q,p}\;\prod_{j=1}^{k}W_{a,b;q,p}\left(j,1\right)+{\left[\begin{array}{c} n\\ k-1 \end{array}\right]}_{aq,bq^{2};q,p}.$$

**Definition** **3.** *Let x,y,a,b be four variables with ab=ba and q,p be two complex numbers with |p|<1. We define $C_{a,b;q,p}\left[x,y\right]$ to be the unital associative algebra over C, generated by x and y, satisfying the following commutation relations*

(17a) $$\begin{array}{cc} yx & =\frac{\theta (aq^{3},bq,a/bq;p)}{\theta (aq,bq^{3},aq/b;p)}qxy, \end{array}$$

(17b) $$\begin{array}{cc} x\;f(a,b) & =f(aq,bq^{2})x, \end{array}$$

(17c) $$\begin{array}{cc} y\;f(a,b) & =f(aq^{2},bq)y, \end{array}$$

*where f(a,b) is any function that is multiplicatively p-periodic in a and b, i.e., which satisfies f(pa,b)=f(a,pb)=f(a,b).*

The relations in (17) are essentially an elliptic realization of the relations in (6). In particular, (17a) can be written as yx=w(1,1)xy with $w\left(s,t\right)=w_{a,b;q,p}\left(s,t\right)$ being the small elliptic weight in (13a).

We refer to the variables x,y,a,b forming $C_{a,b;q,p}\left[x,y\right]$ as *elliptic-commuting* variables. The algebra $C_{a,b;q,p}\left[x,y\right]$ formally reduces to $C_{q}\left[x,y\right]$ if one lets p→0, a→0, then b→0 (in this order), while, having eliminated the nome *p*, relaxing the two conditions of multiplicative *p*-periodicity.

In $C_{a,b;q,p}\left[x,y\right]$ the following binomial theorem holds as a consequence of Theorem 1 (cf. [10]):

(18) $${\left(x+y\right)}^{n}=\sum_{k=0}^{n}{\left[\begin{array}{c} n\\ k \end{array}\right]}_{a,b;q,p}x^{k}y^{n-k}.$$

It is now straightforward to extend $C_{a,b;q,p}\left[x,y\right]$ in the spirit of Definition 2 to an algebra we name $C_{a,b;q,p}\left[x,x^{-1},y\right]$ (and keep referring to as algebra of elliptic-commuting variables).

**Definition** **4.** *Let x,y,a,b be four variables, x invertible, with ab=ba and q,p be two complex numbers with |p|<1. We define $C_{a,b;q,p}\left[x,x^{-1},y\right]$ to be the unital associative algebra over C, generated by x, $x^{-1}$, and y, satisfying the following commutation relations*

(19a) $$\begin{array}{cc} x^{-1}x & =xx^{-1}=1 \end{array}$$

(19b) $$\begin{array}{cc} yx & =\frac{\theta (aq^{3},bq,a/bq;p)}{\theta (aq,bq^{3},aq/b;p)}qxy, \end{array}$$

(19c) $$\begin{array}{cc} x^{-1}y & =\frac{\theta (aq^{2},b/q,a/b;p)}{\theta (a,bq,aq^{2}/b;p)}qyx^{-1}, \end{array}$$

(19d) $$\begin{array}{cc} x\;f(a,b) & =f(aq,bq^{2})x, \end{array}$$

(19e) $$\begin{array}{cc} x^{-1}\;f\left(a,b\right) & =f\left(aq^{-1},bq^{-2}\right)x^{-1}, \end{array}$$

(19f) $$\begin{array}{cc} y\;f(a,b) & =f(aq^{2},bq)y, \end{array}$$

*where f(a,b) is any function that is multiplicatively p-periodic in a and b.*

Again, it is not difficult to see that the conditions in (19) are compatible with each other and naturally extend those in (17) by adding relations involving $x^{-1}$.

## 4. Noncommutative Fibonacci Polynomials

In the following, we shall first assume *x* and *y* (which in Section 3 were prescribed to satisfy specific commutation relations) to be non-commutative variables without any relation connecting them; we shall only later specialize *x* and *y* when explicitly stated.

The material in this section, up to (23), is essentially a review of work done by Johann Cigler and is included here for convenience and self-containedness.

The *noncommutative* (or *abstract*) Fibonacci polynomials of Cigler [6,7] are defined by $F_{0}\left(x,y\right)=1$, $F_{1}\left(x,y\right)=y$ and

(20a) $$\begin{array}{cc} F_{n+2}\left(x,y\right) & =F_{n}\left(x,y\right)x+F_{n+1}\left(x,y\right)y, \end{array}$$

or equivalently

(20b) $$\begin{array}{cc} 1-1F_{n+2}\left(x,y\right) & =x\;F_{n}\left(x,y\right)+y\;F_{n+1}\left(x,y\right), \end{array}$$

for all n≥0.

The equivalence of (20a) and (20b) will be shown later. (See the explanation right after (21).) Combinatorially, $F_{n}\left(x,y\right)$ represents the sum of the weights of all possible ordered tilings of a 1×n board in 1×2 dominoes weighted with *x* and 1×1 squares weighted with *y*. (This also explains why the two recurrences in (20) are equivalent.)

**Example** **1.**

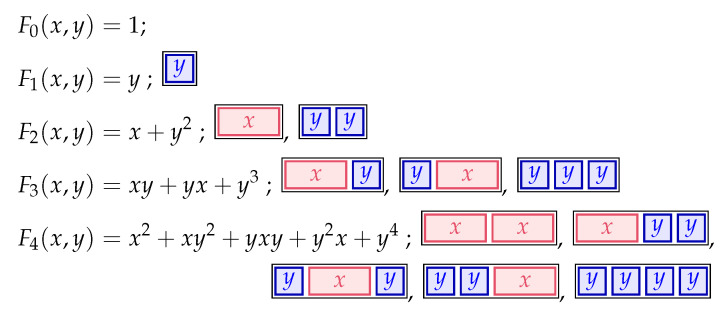

Let

$$C\left(x,y\right):=\left(\begin{array}{cc} 0 & 1\\ x & y \end{array}\right).$$

Then the *n*-th power of the matrix C(x,y) can be expressed nicely in terms of the non-commutative Fibonacci polynomials (as was already shown by Cigler (Equation (3.2) in [7]).

**Proposition** **2.** $$C^{n}\left(x,y\right)=\left(\begin{array}{cc} F_{n-2}\left(x,y\right)x & F_{n-1}\left(x,y\right)\\ F_{n-1}\left(x,y\right)x & F_{n}\left(x,y\right) \end{array}\right),$$

*for n≥2.*

**Proof.**  We proceed by induction.

For n=2,

$$C^{2}\left(x,y\right)=\left(\begin{array}{cc} 0 & 1\\ x & y \end{array}\right)\left(\begin{array}{cc} 0 & 1\\ x & y \end{array}\right)=\left(\begin{array}{cc} x & y\\ yx & x+y^{2} \end{array}\right)=\left(\begin{array}{cc} F_{0}\left(x,y\right)x & F_{1}\left(x,y\right)\\ F_{1}\left(x,y\right)x & F_{2}\left(x,y\right) \end{array}\right).$$

Suppose that

$$C^{n-1}\left(x,y\right)=\left(\begin{array}{cc} F_{n-3}\left(x,y\right)x & F_{n-2}\left(x,y\right)\\ F_{n-2}\left(x,y\right)x & F_{n-1}\left(x,y\right) \end{array}\right)$$

holds for some n−1≥2. Then

$$\begin{array}{cc} C^{n}\left(x,y\right) & =C^{n-1}\left(x,y\right)C\left(x,y\right)\\ \; & =\left(\begin{array}{cc} F_{n-3}\left(x,y\right)x & F_{n-2}\left(x,y\right)\\ F_{n-2}\left(x,y\right)x & F_{n-1}\left(x,y\right) \end{array}\right)\left(\begin{array}{cc} 0 & 1\\ x & y \end{array}\right)\\ \; & =\left(\begin{array}{cc} F_{n-2}\left(x,y\right)x & F_{n-3}\left(x,y\right)x+F_{n-2}\left(x,y\right)y\\ F_{n-1}\left(x,y\right)x & F_{n-2}\left(x,y\right)x+F_{n-1}\left(x,y\right)y \end{array}\right)\\ \; & =\left(\begin{array}{cc} F_{n-2}\left(x,y\right)x & F_{n-1}\left(x,y\right)\\ F_{n-1}\left(x,y\right)x & F_{n}\left(x,y\right) \end{array}\right), \end{array}$$

by (20a). (Similary, we could have used $C^{n}\left(x,y\right)=C\left(x,y\right)C^{n-1}\left(x,y\right)$ instead, in combination with (20b).) □

Since $C^{m+n}\left(x,y\right)=C^{m}\left(x,y\right)C^{n}\left(x,y\right)$, we have

$$\begin{array}{cc} C^{m+n}\left(x,y\right)\; & =\left(\begin{array}{cc} F_{m+n-2}\left(x,y\right)x & F_{m+n-1}\left(x,y\right)\\ F_{m+n-1}\left(x,y\right)x & F_{m+n}\left(x,y\right) \end{array}\right), \end{array}$$

and

$$\begin{array}{cc} 1-1C^{m}\left(x,y\right)C^{n}\left(x,y\right)\; & =\left(\begin{array}{cc} F_{m-2}\left(x,y\right)x & F_{m-1}\left(x,y\right)\\ F_{m-1}\left(x,y\right)x & F_{m}\left(x,y\right) \end{array}\right)\left(\begin{array}{cc} F_{n-2}\left(x,y\right)x & F_{n-1}\left(x,y\right)\\ F_{n-1}\left(x,y\right)x & F_{n}\left(x,y\right) \end{array}\right). \end{array}$$

By comparing the components, we obtain the formula

(21) $$F_{m+n}\left(x,y\right)=F_{m-1}\left(x,y\right)\;x\;F_{n-1}\left(x,y\right)+F_{m}\left(x,y\right)\;F_{n}\left(x,y\right).$$

We proved (21) using the recurrence (20a), which can be recovered from the former by letting (m,n)↦(n+1,1). However, (21) also includes the recurrence (20b), obtained by letting (m,n)↦(1,n+1). This shows that the two recurrences in (20) are indeed equivalent.

From a combinatorial view, the identity (21) is clear. A tiling of the 1×(m+n) board can be split into two independent tilings of lengths *m* and *n*, or there is a domino in the middle, right between two independent tilings of respective lengths m−1 and n−1.

We now use (21) in conjunction with negatively indexed non-commutative Fibonacci polynomials to obtain a non-commutative (Euler–)Cassini identity. In fact, one can simply use the recurrences in (20) to define non-commutative Fibonacci polynomials of negative index. It turns out that these happen to be polynomials in $x^{-1}$ (so we must assume *x* to be invertible). In particular, application of (20) in the negative direction gives

$$\begin{array}{cc} F_{-1}\left(x,y\right) & =0,\\ F_{-2}\left(x,y\right) & =x^{-1},\\ F_{-3}\left(x,y\right) & =-x^{-1}yx^{-1},\\ F_{-4}\left(x,y\right) & =x^{-2}+x^{-1}yx^{-1}yx^{-1},\\ F_{-5}\left(x,y\right) & =-x^{-2}yx^{-1}-x^{-1}yx^{-2}-x^{-1}yx^{-1}yx^{-1}yx^{-1},\\ F_{-6}\left(x,y\right) & =x^{-3}+x^{-2}yx^{-1}yx^{-1}+x^{-1}yx^{-2}yx^{-1}\\ \; & \;\;+x^{-1}yx^{-1}yx^{-2}+x^{-1}yx^{-1}yx^{-1}yx^{-1}yx^{-1}. \end{array}$$

It is easy to use (20) and induction to prove that

(22) $$F_{-n}\left(x,y\right)={\left(-1\right)}^{n}F_{n-2}\left(x^{-1},x^{-1}y\right)\;x^{-1},$$

for all integers *n*. The formula (22) was also obtained by Cigler (Equation after (3.4) in [7]).

We can also use matrices to arrive at negatively indexed noncommutative Fibonacci polynomials, namely

$$C^{-1}\left(x,y\right)=\left(\begin{array}{cc} -x^{-1}y & x^{-1}\\ 1 & 0 \end{array}\right),$$

which satisfies $C^{-1}\left(x,y\right)C\left(x,y\right)=C\left(x,y\right)C^{-1}\left(x,y\right)=I_{2}$; Proposition 2 is easily seen to extend to all integers *n* with the negatively indexed noncommutative Fibonacci polynomials (defined recursively by (20), and which can be expressed by the non-negatively indexed noncommutative Fibonacci polynomials by (22)). This was actually how Cigler arrived at (22) in [7]. This shows that (21), which we originally proved for positive integers *m* and *n*, actually holds for all integers *m* and *n*.

We now let m=−n in (21) and multiply both sides of the identity by ${\left(-1\right)}^{n}$ and arrive, after two applications of (22) at the *non-commutative Cassini identity* (cf. Section 3 in [7])

(23) $${\left(-1\right)}^{n}=F_{n-2}\left(x^{-1},x^{-1}y\right)\;x^{-1}\;F_{n}\left(x,y\right)-F_{n-1}\left(x^{-1},x^{-1}y\right)\;F_{n-1}\left(x,y\right),$$

which is valid for all integers *n*.

More generally, we may take (m,n)↦(−n,n+k) in (21) and multiply both sides of the identity by ${\left(-1\right)}^{n}$ and arrive, after two applications of (22), at the *non-commutative Euler–Cassini identity*

(24) $${\left(-1\right)}^{n}F_{k}\left(x,y\right)=F_{n-2}\left(x^{-1},x^{-1}y\right)\;x^{-1}\;F_{n+k}\left(x,y\right)-F_{n-1}\left(x^{-1},x^{-1}y\right)\;F_{n+k-1}\left(x,y\right),$$

which is valid for all integers *n* and *k*.

**Remark** **1.** *We would like to mention that in the classical case the Cassini identity is usually obtained by taking the determinants of the n-th power of the Fibonacci matrix $\begin{array}{c} \left(\begin{array}{cc} 0 & 1\\ 1 & 1 \end{array}\right) \end{array}$ and using the property that the determinant of matrices with commuting entries is multiplicative. This method to obtain a Cassini identity requires adaptation in the non-commutative setting since the determinant is in general not multiplicative if the matrices contain entries that do not commute. In some special cases (in particular when considering quantum matrix representations of quantum groups) this can be remedied by suitably modifying the definition of determinant and by considering* quantum determinants *instead where the entries of the matrices obey certain commutation relations.*

*We can apply this construction with the necessary adaptions here as well. For four polynomials $a\left(x,x^{-1},y\right)$, $b\left(x,x^{-1},y\right)$$c\left(x,x^{-1},y\right)$$d\left(x,x^{-1},y\right)$ in the non-commuting variables x, $x^{-1}$ and y (with $x^{-1}x=xx^{-1}=1$), over some ground field K (say K=C), define the noncommutative determinant $\vec{\det}$ by*

(25) $$\begin{array}{cc} \; & \vec{\det}\left(\begin{array}{cc} a\left(x,x^{-1},y\right) & b\left(x,x^{-1},y\right)\\ c\left(x,x^{-1},y\right) & d\left(x,x^{-1},y\right) \end{array}\right)\\ & :=a\left(x^{-1},x,x^{-1}y\right)\;d\left(x,x^{-1},y\right)-c\left(x^{-1},x,x^{-1}y\right)\;x\;b\left(x,x^{-1},y\right). \end{array}$$

*Now $\vec{\det}$ is in general not multiplicative but for suitable choices of the matrices it is. This in particular applies to matrices given by any integer power of C(x,y), as one can easily verify. Now taking the noncommutative determinant $\vec{\det}$ of $C^{n}\left(x,y\right)$ and comparing it with the n-th power of $\vec{\det}\;C\left(x,y\right)=-1$, we readily obtain the non-commutative Cassini identity (23).*

### 4.1. Noncommutative Weight-Dependent Fibonacci Polynomials

We consider the noncommutative Fibonacci polynomials $F_{n}\left(x,y\right)$ with additional weight-dependent commutation relations imposed to involve a doubly indexed sequence of invertible weights ${\left(w\left(s,t\right)\right)}_{s\in Z,t\in N}$. More precisely we shall work in the algebra of weight-dependent variables $C_{w}\left[x,x^{-1},y\right]$ defined in Definition 2. We write $F_{n}\left(x,y\;|\;w\right)$ for the respective noncommutative weight-dependent Fibonacci polynomials in this case.

Any expression *X* in $C_{w}\left[x,x^{-1},y\right]$ can be *normalized* and written uniquely as a formal sum (with finitely many non-vanishing terms)

$$X=\sum_{k=-\infty }^{\infty }\sum_{\ell =0}^{\infty }c_{X}\left(k,\ell \right)x^{k}y^{\ell },$$

with $c_{X}\left(k,\ell \right)$ a polynomial expression over C in the $w{\left(s,t\right)}^{\pm 1}$, s∈Z,t∈N. We say that the variables occurring in an expression in $C_{w}\left[x,x^{-1},y\right]$ have been *normally ordered* if, as above, in each of the monomials all the occurrences of *y* have been moved (with the help of commutation relations, if necessary) to the most right, followed by the occurrences of *x* or $x^{-1}$ to the left (again with the help of commutation relations, if necessary) and if only to the most left polynomials of the respective monomials the various weights $w{\left(s,t\right)}^{\pm 1}$ appear.

From the first recurrence relation in (20) and the recurrence for the weighted binomial coefficients in (8) (where the “big weight” W(s,k) that appears there is a product of small weights w(s,t), according to (7)) we can easily prove the following result for the normally ordered noncommutative weight-dependent Fibonacci polynomials.

**Proposition** **3.** *As elements of $C_{w}\left[x,x^{-1},y\right]$, the noncommutative weight-dependent Fibonacci polynomials $F_{n}\left(x,y\;|\;w\right)$ (which are recursively defined by the two intial values $F_{0}\left(x,y\;|\;w\right)=1$, $F_{1}\left(x,y\;|\;w\right)=y$, and either one of the two recurrence relations in *(20)*) take the following normalized form:*

(26) Fn(x,y|w)=∑k=0nwwwn−kkxkyn−2k,

*where wwwnk is the weight-dependent binomial coefficient recursively defined in (8), for any non-negative integer n.*

**Proof.**  We proceed by induction. For n=0 and n=1 (26) is clear. Now assume that the formula is true for all non-negative integers up to n+1. To show it for the next value, n+2, apply the first identity in (20) to split $F_{n+2}\left(x,y\;|\;w\right)$ in two lower-indexed noncommutative weight-dependent Fibonacci polynomials and apply the induction hypothesis. Concretely, we have

Fn+2(x,y|w)=Fn+1(x,y|w)y+Fn(x,y|w)x=∑k=0n+1wwwn+1−kkxkyn+1−2k·y+∑k=1n+1wwwn−(k−1)k−1xk−1yn+2−2k·x=∑k=0n+1wwwn+1−kkxkyn+1−2k·y+∑k=1n+1wwwn−(k−1)k−1xk−1W(1,n+2−2k)xyn+2−2k=∑k=0n+2wwwn+1−kk+wwwn+1−kk−1W(k,n+2−2k)xkyn+2−2k=∑k=0n+2wwwn+2−kkxkyn+2−2k,

where we have applied an instance of Lemma 1 in the third equality and the recursion for the weight-dependent binomial coefficients (8) in the last equality. □

A great deal of the analysis from the beginning of this section which concerned the noncommutative Fibonacci polynomials extends to the noncommutative weight-dependent case without much changes. This in particular concerns the formula (21) which now obviously takes the form

(27) $$F_{m+n}\left(x,y\;|\;w\right)=F_{m-1}\left(x,y\;|\;w\right)\;x\;F_{n-1}\left(x,y\;|\;w\right)+F_{m}\left(x,y\;|\;w\right)\;F_{n}\left(x,y\;|\;w\right).$$

Again, this formula holds for all integers *m* and *n* but we still have to specify the exact form of the negatively indexed noncommutative weight-dependent Fibonacci polynomials. By carrying out the same analysis that led to (22) (i.e., application of the recurrence (20) in the negative direction, and induction) adapted to the weight-dependent setting, we obtain

(28) $$F_{-n}\left(x,y\;|\;w\right)={\left(-1\right)}^{n}F_{n-2}\left(x^{-1},x^{-1}y\;|\;\tilde{w}\right)\;x^{-1},$$

where the dual weight function $\tilde{w}$ is defined in (12).

The noncommutative weight-dependent Euler–Cassini identity thus takes the following form:

(29) $$\begin{array}{cc} {\left(-1\right)}^{n}F_{k}\left(x,y\;|\;w\right) & =F_{n-2}\left(x^{-1},x^{-1}y\;|\;\tilde{w}\right)\;x^{-1}\;F_{n+k}\left(x,y\;|\;w\right)\\ \; & \;\;-F_{n-1}\left(x^{-1},x^{-1}y\;|\;\tilde{w}\right)\;F_{n+k-1}\left(x,y\;|\;w\right), \end{array}$$

which is valid for all integers *n* and *k*.

### 4.2. Noncommutative Elliptic Fibonacci Polynomials

We now specialize the weights $w\left(s,t\right):=w_{a,b;q,p}\left(s,t\right)$ (for s∈Z and t∈N) to be the elliptic weights defined in (13a), where a,b are two independent parameters and p,q are complex numbers with |p|<1. We are thus working in the algebra of elliptic-commuting variables $C_{a,b;q,p}\left[x,x^{-1},y\right]$ defined in Definition 4. We write $F_{n}\left(x,y\;|\;a,b;q,p\right)$ for the respective noncommutative elliptic Fibonacci polynomials in this case.

Specialization of Proposition 3 readily gives the following result.

**Corollary** **1.** *As elements of $C_{a,b;q,p}\left[x,x^{-1},y\right]$, the* noncommutative elliptic Fibonacci polynomials*$F_{n}\left(x,y\;|\;a,b;q,p\right)$ take the following normalized form:*

(30) $$F_{n}\left(x,y\;|\;a,b;q,p\right)=\sum_{k=0}^{n}{\left[\begin{array}{c} n-k\\ k \end{array}\right]}_{a,b;q,p}x^{k}y^{n-2k},$$

*where ${\begin{array}{c} \left[\begin{array}{c} n\\ k \end{array}\right] \end{array}}_{a,b;q,p}$ is the elliptic binomial coefficient given in *(16)*, for any non-negative integer n.*

Now, the specialization of (27) is straightforward and gives

(31) $$\begin{array}{cc} F_{m+n}\left(x,y\;|\;a,b;q,p\right) & =F_{m-1}\left(x,y\;|\;a,b;q,p\right)\;x\;F_{n-1}\left(x,y\;|\;a,b;q,p\right)\\ \; & \;\;+F_{m}\left(x,y\;|\;a,b;q,p\right)\;F_{n}\left(x,y\;|\;a,b;q,p\right), \end{array}$$

which again holds for all integers *m* and *n*.

Finally, we determine the exact form of the negatively indexed noncommutative elliptic Fibonacci polynomials. Combination of (12) and (15) gives the following formula for the *dual weights*

(32) $${\tilde{w}}_{a,b;q,p}\left(s,t\right)=w_{a/b,1/b;q,p}\left(s,t\right),$$

which shows that the negatively indexed noncommutative elliptic Fibonacci polynomials can be conveniently written in terms of the non-negatively indexed ones. We thus have

(33) $$F_{-n}\left(x,y\;|\;a,b;q,p\right)={\left(-1\right)}^{n}F_{n-2}\left(x^{-1},x^{-1}y\;|\;a/b,1/b;q,p\right)\;x^{-1}.$$

The noncommutative elliptic Euler–Cassini identity thus takes the following form:

(34) $$\begin{array}{cc} {\left(-1\right)}^{n}F_{k}\left(x,y\;|\;a,b;q,p\right)\; & =F_{n-2}\left(x^{-1},x^{-1}y\;|\;a/b,1/b;q,p\right)\;x^{-1}\;F_{n+k}\left(x,y\;|\;a,b;q,p\right)\\ \; & \;\;-F_{n-1}\left(x^{-1},x^{-1}y\;|\;a/b,1/b;q,p\right)\;F_{n+k-1}\left(x,y\;|\;a,b;q,p\right), \end{array}$$

which is valid for all integers *n* and *k*.

## Data Availability

Not available.
